# Preferences for index-based crop insurance in South Africa

**DOI:** 10.4102/jamba.v16i1.1611

**Published:** 2024-06-12

**Authors:** Phiwe Jiba, Mzuyanda Christian, Khulekani Nxumalo, Victor Mmbengwa

**Affiliations:** 1 Department of Agricultural Economics and Extension, Faculty of Natural and Agricultural Sciences, North-West University, Mmabatho, South Africa; 2 School of Agribusiness, Faculty of Agricultural Economics, Nelson Mandela University, Port Elizabeth, South Africa; 3 Department of Agricultural Economics and Extension, Faculty of Agricultural Science, University of Limpopo, Mankweng, South Africa

**Keywords:** index-base insurance, probit model, willingness to participate, natural hazards, Eastern Cape

## Abstract

The main reason cited for the low participation rate in indexed insurance in Africa is the lack of farmers’ involvement in the initial conception. The main aim of this article was, therefore, to design an improved participatory methodology that would help farmers in South Africa gather information on the value they place in new crop insurance initiatives based on weather indices. The main objective of this study was to determine their willingness to pay (WTP) crop insurance in the Eastern Cape. The study adopted a cross-sectional design, and the respondents were selected by using a probability sampling procedure. A sample of 150 respondents was selected. The results from descriptive statistics showed that 62% of the smallholder irrigation scheme farmers were between 52 and above years of age, male and female: 87% and 13%, respectively. The results from the probit model revealed that out of nine variables included in the model, only four namely, logAge square, gender, marital status and age were significantly influencing the WTP index-based crop insurance at 1%, 5% and 10%, respectively. However, age was found to reduce the chances of participating in crop-based insurance. A large proportion of those who were willing to pay insurance had not previously experienced losses because of natural hazards. Therefore, it recommended that the government should use existing extension officers to educate the farmers and promote the importance and benefits of insurance products.

**Contribution:**

The results are expected to contribute to the local government to improve disaster resilience through strengthening regional financial funding.

## Introduction

South Africa’s agricultural sector is vital to its economy, contributing significantly to employment and gross domestic product (GDP). However, the sector faces numerous hazards that threaten crop production and food security. Understanding these hazards and their relative significance is crucial for developing effective mitigation strategies and ensuring the resilience of the agricultural sector. Agricultural insurance is a type of coverage that offers financial protection to farmers in case of loss of crops, livestock or income because of unforeseen events such as natural hazards, pests or diseases. This type of insurance is designed to help farmers recover from losses and keep their farming operations running smoothly. With agricultural insurance, farmers can have peace of mind knowing that they will be compensated for their losses, which can help them to stay afloat during difficult times. (Joshua et al. 2019). It also protects individuals exposed to production or other risks with the ability to plan for future unexpected losses. Crop insurance has become an important adaptive tool for managing economic and adverse environmental risks in the agricultural sector (Fahad et al. 2018). Moreover, changes can introduce shocks to the agricultural sector. Natural disasters, evolving demand patterns, technological advancements, and overall economic and agricultural policies can impact economies globally. These changes can be integrated into the context of agricultural production (Van Zyl, Nel & Groenewald 2010).

Farmers can often benefit from investments in agricultural activities that require higher initial investments but are ultimately more profitable if those investments carry risks such as: generating high profits. Banks and other intermediaries working with agricultural producers bear the same risks as their agricultural customers, so they too may be reluctant to invest in agriculture as they may default during or after a natural hazard such as a weather event. Risk management strategies involving risk sharing include but are not limited to agricultural financing, leasehold and prize pool agreements, agricultural futures contracts and future market hedging. In addition, agricultural insurance can be an important means of transferring some of these risks (Mersha 2001).

According to Norton et al. (2012), weather index insurance is a promising tool for transferring risk from rural communities to global insurance pools. Index insurance refers to insurance policies based on an index of weather variables such as rainfall for drought insurance that acts as a proxy for crop yield. The impacts of climate change and climate change-related agriculture are immense (Sibiko 2016). For example, rising temperatures and unpredictable seasonal rainfall are already adversely affecting global grain production, especially in vulnerable regions of South Asia and Africa. Norton et al. (2012) further argue that insurance contracts are based on objectively observed weather variables in the region, a costly and potentially subjective adjustment process. It avoids some of the shortcomings of the base insurance product. In developing countries, a single contract can cover all local farmers, even if it is prohibitively expensive to adapt to individual farmland conditions.

The general problem is the perceived high risks faced by smallholder irrigation schemes on crop production, transactions and human resources which often affect their farming operations as well as their livelihoods. Regardless of the affordability aspect of South African crop insurance, currently available insurance products include Multi-Peril Crop Insurance (MPCI) and Hail Plus Coverage. Therefore, the probit regression model was used to analyse the main problem of the study. Importantly, this study contributes to the growing literature on the uptake of index-based crop insurance in South Africa and classifies the key challenges driving the significant rescaling and expansion of this insurance plan as a climate change adaptation strategy.

### Status quo on the use of agricultural insurance by farmers

According to Nieuwoudt (2000), South Africa’s attempt to broaden access to drought-inclusive insurance was not particularly successful. In general, agricultural insurance is one method by which farmers can stabilise farm income and investment from the terrible effect of loss because of natural hazards or low market prices. In recent years, South African agriculture has become a volatile environment that is mainly influenced by climate conditions and economic instabilities, risks and uncertainty are intrinsic to the farming environment. The main uncertainty that the South African farming environment is currently facing is the implementation of policies, specifically the land reform policies (Scheepers 2017). The land reform and redistribution in South Africa have resulted in new farmers struggling to own their land and therefore not having collateral to use as required for access to financial credit.

### Empirical studies on the benefit of adopting agricultural insurance

According to Zhao et al. (2016), agricultural insurance helps to reduce the disparity in losses among farmers, and through subsidies, the programme provides farmers with income transfers from other parts of the economy. Liu (2016) found that the introduction of crop insurance was positively correlated with natural hazard impact, level of insurance coverage, level of yield stability, level of government subsidies and farmers’ trust in the government. Zhao et al. (2016) further observed that insurance is an important part of agricultural insurance programmes and governments use insurance as a useful tool aimed at stabilising the agricultural sector through income stabilisation and increasing production over the long term, and they see it as a viable approach. Ntukamazina et al. (2017) advocated income stabilisation through agricultural insurance, adding that agricultural insurance not only stabilises income but also helps farmers resume production activities after poor harvests. Insurance can crowd out farmers’ demand for future prices (Li et al. 2013). Despite this possible crowding-out effect, there is little literature on this topic that empirically examines farmers’ actual responses to crop insurance when making pricing decisions.

Contrary to the above endorsement, Leblois and Quirion (2013) argued that the income impact of these farm insurance programmes could not be estimated because of the favourable rainy season during which they were implemented. Use of costly inputs increased year-on-year, but surprisingly insurance had no positive impact on loan use of inputs. Hazell and Hess (2010) added that climate risk poses a high level of agricultural production risks that threaten household income, food security and debt repayment. Additionally, Eza et al. (2020) mentioned that weather index insurance is also able to assist in improving food security for smallholder farmers in drought conditions and encourage higher production in years without drought. Leblois and Quirion (2013) also found that post-educated policy misconceptions may be because of inadequate educational attainment. According to Li et al. (2013), insurance can crowd out farmers’ demand for forward pricing. Sumani (2018) argues that the agricultural insurance industry can make a positive contribution to creating carbon sinks, sequestering carbon and further reducing greenhouse gas emissions, provided that appropriate policy and regulatory frameworks are well established. Reported option contract effects vary depending on the term and type of crop insurance. However, this implies that nothing will even be at the maximum required level to achieve the regulatory goals as there will be a trade-off between politics and other socio-economic considerations. Conversely, Eza et al. (2020) view Weather Index Insurance (WII) as derivatives not insurance, which is based on environmental variables and not actual losses. Although agricultural insurance has failed in South Africa, Busnita et al. (2012) mentioned that some countries have implemented agriculture insurance and proved to be successful, and it is the latest innovation offered to protect farmers from the threat of failed harvest.

Because of the large number of ranchers affected by natural hazards, doubts about weather conditions and weather-related hazards can cause significant economic damage. Livestock insurance is therefore the best way to mitigate economic losses for livestock farmers (Kairala & Bhandari 2018). In addition, index insurance can be used for a variety of weather-related risks, from crop loss because of natural hazards such as droughts and floods, to livestock loss because of severe cold weather and storm damage (Hellmuth et al. 2009).

### Advantages of index-based weather insurance

Compared to traditional agricultural insurance where indemnity payments are made based on individual losses, index-based weather insurance has advantages in low transaction costs associated with asymmetric information and administration costs as well as a simple standard and transparent structure. Moral hazard should be low as the insured should not be able to influence the outcome of the index. The indemnity payments for index-based insurance are not influenced by another factor. Furthermore, adverse selection should be much lower as insurers do not need to have a great deal of information about risk exposures for the individual policyholders, and individual risk exposure does not impact their risk classes. Finally, as there is no loss adjustment, the transaction cost should also be lower if there are existing systems to establish the basis of the index payments. All these factors should lower the administrative cost of the insurance.

### Previous related studies on agricultural crop insurance

Several studies were carried out in different regions, using different methods to determine the farmer’s willingness to pay (WTP) for crop insurance. Ali (2013) assessed farmers’ WTP for insurance in the rain-fed areas of Pakistan using the probit model to estimate the farmers’ willingness to join and the acreage farmers are interested to ensure is estimated by employing the Poisson regression estimates. Ali (2013) then used propensity score matching to estimate the likelihood of the impact of insurance. The results showed that farmers’ economic status, household assets and membership in community organisations are crucial determinants of their WTP a higher insurance premium. The propensity scores matching results have indicated that farmers were satisfied with index-based insurance and were also willing to increase the area to plant.

Shashi Kiran and Umesh (2015) further estimate the WTP by rainfed maize farmers for crop insurance premiums and the factors influencing their WTP. To estimate the WTP, the Double-Bounded Dichotomous Choice method of Contingent Valuation (CV) was used. The results showed that farmers were willing to pay 0.34% more premium to ensure their crop, and the average probability of WTP of farmers for crop insurance premium was 0.53. It was also found that age was the critical factor influencing their WTP, and farmers’ awareness about the products and procedures of crop insurance was poor.

A study in Ghana was carried out to assess the farmers’ WTP for crop insurance by Elum, Modise and Marr (2017). The study employed descriptive statistical methods to analyse the obtained data and found that 52.9% of the farmers were interested in crop insurance. A Heckman two-stage approach was used to estimate the factors that influenced the WTP for crop insurance by farmers using the probit model and found that factors such as education, age, type of crop and income had more influence on the WTP.

## Research methodology

### Description of the study area

The study area mainly covered three districts of the Eastern Cape province. Some irrigation systems were by then operational, while others were being reinstated as part of the government programmes to strengthen the smallholder sector. The study was conducted in the Eastern Cape province, with a population of 6 562 053 (12.7%), the third largest province in South Africa, after Gauteng and KwaZulu-Natal with an estimated population of 12 272 263 million (23.7% of national) and 10 267 300 million (10.8%), respectively. The province consists of eight districts and 42 metropolitan municipalities. Therefore, the study was conducted in three districts of the province with fully functioning irrigation systems for smallholder farmers. The three districts’ municipalities were O.R Tambo, Chris Hani and Amathole District municipality, and one municipality was chosen for each District, namely King Sabata Dalindyebo in O.R Tambo, Intsika Yethu in Chris Hani and Izanyokhwe in Amathole.

This study focused on estimating the WTP for index-based crop insurance of irrigation programme beneficiaries in rural areas of selected districts of the Eastern Cape province, considering various irrigation schemes. The study was based on a cross-sectional design. The survey was compiled based on questionnaires distributed to irrigation users and data were also collected from nearby survey sites. A quantitative method research approach was adopted for the proposed study. Purposive sampling is a sampling technique in which researchers use discretion to select members of a target population to include in the sample. Multistage methods then allow studies to combine stages of the sampling procedure, using three of them (targeted, stratified and random sampling).

Villages were selected randomly according to the availability of irrigation schemes in each municipality. A multi-stage sampling procedure was employed by which irrigation scheme beneficiaries in the Eastern Cape were purposively selected. A total number of 150 irrigation scheme beneficiaries were obtained from all selected municipalities using probability proportional to size. During data collection, a structured questionnaire was distributed to respondents in the form of personal interviews. A quantitative approach was used to collect data using a self-administered questionnaire.

Descriptive refers to a concise collection of statistical measures that provide a summary of a given dataset. The study also aims at examining those factors that influenced irrigation schemes, and the probit regression model was used to analyse willingness to participate in exponential crop insurance. Based on the following equation, the response variable Y is binary. That means one can have two possible outcomes. *β* denotes 0 for those not willing to join and 1 for those who were willing to join agricultural insurance, for example, Y will represent whether one was willing or not to join agricultural insurance by irrigation schemes. The vector of regressor X was assumed to influence the outcome Y. Specifically, we can assume that the model takes this form (see Equations 1 and 2):

$$Pr(Y=1|X)=\emptyset (X^{I}\beta )$$ [Eqn 1]

$$P(Y_{i}=1)=P(Y_{i}^{*}\geq 0)$$ [Eqn 2]

- **Pr(Y = 1| X):** This represents the probability that the dependent variable Y takes on a value of 1 given the values of the independent variable X. In other words, it represents the likelihood of an event (Y = 1) occurring based on certain conditions (values of X).
- **Ø:** This typically represents a function, often used in logistic regression, called the logistic function or sigmoid function. It transforms the linear combination of the independent variables (X) and coefficients (*β*) into a probability value between 0 and 1.
- **(X^I*β*):** This represents the linear combination of the independent variables (X) and coefficients (*β*), where X^I denotes the matrix of independent variables and *β* denotes the vector of coefficients.

where *Pr* denotes the probability, *i* is 1, 2, 3…. n, *β* a factor of unknown coefficients, Ø is the cumulative distribution function (CDF) of the standard normal distribution and *Y* is the probability whether the irrigation scheme is willing to join agricultural insurance: 1 = willingness to join and 0 = not willing to join.

## Results and discussion

### Distribution of irrigation scheme beneficiaries by education

The results show that beneficiaries of irrigation schemes were well-informed and attended lower secondary and higher education, 30% and 26%, respectively. The results show that the educational attainment of these beneficiaries could have helped many of them gain more opportunities in agribusiness, and ultimately, the entire agricultural industry. In addition, higher education had the potential to lift individuals or farmers out of poverty, if the education system could be accessed by the right mini-irrigations at the right time and with the right content. Moreover, higher education provides farmers with access to knowledge and skills that can improve their agricultural practices. This includes learning about new technologies, sustainable farming methods and effective resource management. By applying these practices, farmers can increase their crop yields and productivity, leading to higher incomes and reduced poverty.

### Distribution of beneficiaries in irrigation according to household income

Agricultural income includes income from the sale of agricultural products and non-farm sources such as pensions, remittances, wages and salaries, and others. Results show that many respondents earned R1000 or less for waterers and non-irrigators. The results show that agriculture is also an important source of income, confirming its importance as a contributor to household income. A similar study by Tekana and Oladele (2011) showed that agriculture plays an important role in poverty reduction and food security in rural areas. Furthermore, agriculture alone is not an appropriate source of income for all farmers, regardless of farm size.

### Distribution of beneficiaries in the irrigation perimeter by age

Older farmers will behave differently from younger farmers regarding actions that promote or hinder the transition from home gardening to market-oriented agriculture based on irrigation use. According to the Food and Agricultural Organization (FAO 1983), age affects farmers’ preferences and attitudes. Likewise, the way a person is treated often affects their age; young people may be treated with less respect than older people. The findings indicate that respondents aged 52 years and older received the highest rating at 62%, while those born in 1990 or earlier received the lowest rating at 9%. The results also show that most of the respondents were old and mature. However, most retired people or retirees usually start farming in rural areas to consume or sell their produce.

### Distribution of household size

Substantial difference between the percentage value of the highest and lowest size of the household as indicated by 63%, ranging between one and five members of the household and 3%, ranging between 15 and 20 members of the household. A large family has many different jobs, be it children, adults, family elders or family members. Labour is one of the most important factors of production for smallholder farmers, as they are labour-intensive rather than capital-intensive. Lastly, the discussion of farm size concerning insurance should consider the complex interplay between farm size, risk management strategies, affordability and government policies. Understanding these dynamics can help policymakers and insurance providers tailor their offerings to meet the diverse needs of farmers across different farm sizes.

### Distribution of household heads by gender

More men responded than women, 87% and 13%, respectively. The results show that in most cases, men still lead when it comes to hard labour in some cases. A similar study carried out by Samah (2010) showed that, in agriculture, women are said to have a negative attitude towards agricultural contracts compared to men.

### Distribution of household heads by occupation

The information was searched for both the head of the household and other members of the household and the question was worded openly so that respondents could mention whatever they wanted or not. The results suggest that family members were mainly engaged in, for example, the occupational categories mentioned by respondents included agriculture, paid employment (including a wide range of activities such as domestic help, caretakers, storekeepers etc.), and the unemployed and retired.

### Distribution of awareness of agricultural insurance

Crop and livestock performance depends on biological processes that are influenced by weather, pests and diseases. According to Kahan (2013), pest and disease outbreaks can also significantly reduce crop and livestock yields. But they must choose between growing crops and raising livestock. Resources they expend in tilling crops, planting, fertilising or tending livestock may not be recovered. Because of this, there are risks. Farmers are producing without complete certainty about what will happen to their production.

**FIGURE 1:**
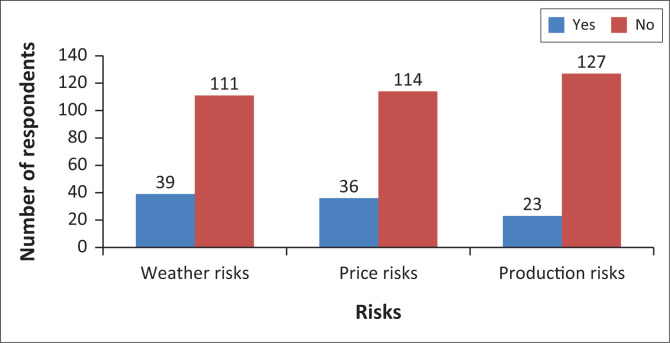
Awareness of agricultural insurance.

Farmer’s awareness about crop-based insurance on weather risks, price risks and production risks schemes was assessed, and the findings of the study in Figure 1 revealed that there were very few smallholders’ irrigation schemes, who were aware of the crop insurance in all the above categories and the maximum value of those, who were aware of the crop insurance was at 40%. In the case of detailed insurance procedures, many of them were not aware of crop-based insurance. This suggests that there was a need to educate irrigation schemes about the existence of crop insurance premiums that were affordable.

### Distribution of farm hazard experience and insurance participation

This shows that most farmers, who never experienced agricultural hazards were generally willing to participate in crop insurance, only a few farmers never experienced any form of agricultural hazards and were not willing to participate in insurance crop danger. Considering the necessity of engaging with knowledgeable farmers who are receptive to new ideas and willing to respond positively to crop insurance incentives. According to Kandel (2019), hazard risk management is crucial for agricultural development in developing countries.

Furthermore, crop insurance indicates a smallholder’s capacity and ability to process and interpret information, helping to better understand and reduce production risk costs. Agricultural insurance plays an important role in agriculture and is an important variable for agricultural decisions in the event of hazards for a particular small farm. Furthermore, Tlholoe (2017) surmises that the more experienced farmers are, the more aware they are of different risk management strategies and thus a lower desire to purchase insurance.

The results obtained from Table 2 show that out of nine variables included in the model, only four variables: logAge^2, gender, marital status and age, significantly influenced WTP for index-based crop insurance at 1%, 5% and 10%, respectively. However, age reduced the participation rate of crop-based crop insurance in this study area. This suggests that the older we are, the more motivated we are to insure them.

**TABLE 1:** Distribution of farm hazards experience and insurance participation.

Hazards	Willing to pay crop insurance		Total
	Yes	No	
Yes	49	19	68
No	73	9	82
**Total**	**122**	**28**	**150**

**TABLE 2:** Factors influencing smallholder’s irrigation schemes participation in crop insurance (*N* = 150).

Willingness to pay (dependent variable)	Coef. std.	Std. error	*z*	*P* > *z*	95% Conf. Interval
Constant	−7.129269	3.176719	−2.24	0.025	−13.35552 – −0.9030135
Agesquare	0.0002589	0.000261	0.99	0.321	−0.0002526 – 0.0007704
Credit	0.3906227	0.2671649	1.46	0.144	−0.133011 – 0.9142563
Extension officers	0.2985244	0.2821581	1.06	0.290	−0.2544953 – 0.8515441
Farming experience	−0.0472563	0.0312126	−1.51	0.130	−0.1084318 – 0.0139193
Number of households	0.0696812	0.066673	1.05	0.296	−0.0609956 – 0.2003579
Age	−0.0568444	0.243301	−2.34	0.019	−0.1045304 – −0.0091584
Gender	0.8053198	0.3444987	2.34	0.019	0.1301147 – 1.480525
Marital status	0.4984847	0.2755108	1.81	0.070	−0.0415065 – 1.038476
logAge2	0.9839613	0.4944804	1.99	0.047	0.0147974 – 1.953125

Note: LR Chi^2^ (9) = 24.46; Prob > Chi^2^ = 0.0036; Pseudo *R*^2^ = 0.1694; Probit regression: Log Likelihood = −59.973985.

Coef. std., coeficient standard; Std. error, standard error; Conf. Interval, confidence interval.

Younger heads of beneficiaries in irrigated areas are more active in adopting innovations than older heads of households; however, in general, they are busy with employment opportunities other than agriculture. This implies that older members of the household are said to have more farming experience and therefore an increased likelihood of insurance coverage based on the crop index, as shown by a positive response of logAge^2.

Gender played a significant role in determining the participation of beneficiaries from small irrigation schemes in crop insurance, with both male and female-headed households having the option to participate or abstain. Likewise, the results were consistent with the findings of Tekana and Oladele (2011), who stated that male-headed households experienced significant improvements in their welfare from farming irrigated industry.

Marital status was positively significant with willingness to adopt index-based crop insurance. This conformed to the *a priori* expectation and is also consistent with other studies (Ellis 2017). Married farmers have the responsibility of reducing their household’s vulnerability to risks and the resulting negative impacts and are therefore more likely to purchase a crop insurance policy.

Farm credit makes rural households more likely to have farm insurance. However, Gulseven’s results (2014) indicate that a significant proportion of farmers do not believe smallholder insurance is necessary. This belief is a serious drawback in smallholder insurance. As incomes increase, the price gap may narrow, leading to a reduced need for insurance subsidies. Insurance demand is a common need among farmers.

## Conclusion and recommendation

The context of the study was seriously discussed, focusing on the international level and then it also focused on Africa as a whole, and finally, focused on South Africa. Based on critical context, the problem statement is defined and addressed by the overall goal of the research, leading to the specific objectives, research questions, hypotheses and importance of the research.

The common problem is the high risks that irrigation facilities face to agricultural production, transactions and human resources, often affecting their agricultural activities as well as their livelihoods. Despite the affordability of crop insurance in South Africa, insurance products available include Multi-Dangerous Crop Insurance (MPCI) and Hail Plus Insurance. These products are quite expensive for small farmers. Furthermore, index-based crop insurance is the only solution or mechanism available to protect farmers against production risks. Furthermore, despite the many government initiatives and benefits and the support of government sectors, the adoption of index-based crop insurance is very low and previous research has not adequately addressed the environmental conditions of man-made disasters, natural hazards and stress. Rather, the majority of studies on index-based crop insurance demand have concentrated on individual crop insurance.

Based on the findings of this study, it is recommended to conduct seminars and research on trust factors in the design and implementation of index-based crop insurance. This will not only enhance local farmers’ understanding of insurance policies but will also create a platform for farmers to make recommendations on insurance design. Government intervention in index-based crop insurance will help to allow for increased affordability for smallholder farmers, allowing most farmers to be able to hedge against risks that threaten their crop production.

### Determinants of demand to purchase index-based crop insurance

Government interventions through premium subsidisation would play a bigger role in increasing the farmer’s demand for index-based crop insurance. Based on the findings that farmers are willing to participate in crop insurance if the premiums are paid post-harvest with proceeds from the crop, the government has opted for index-based crop insurance by granting grants to sponsors.

### Ability to pay premiums by irrigation scheme beneficiaries

Premium subsidies can keep premium rates sustainable for insurers. Insurance companies are often forced to reduce premium rates to increase insurance demand. Institutional development, including providing market information, developing contractual arrangements and institutional support, should be considered. This can be accomplished through farmer group marketing arrangements as they can reduce transportation costs to the market and increase output to the market. The government can also support this arrangement by investing in public facilities such as markets and telecommunications. Farmers need better training to evaluate the risk management strategies that best match natural risk management. Therefore, the study recommends promoting better farmer education programmes and educating them on how to evaluate risk management tools.

### Areas to be focused on for future

Despite the significant role that insurance plays in agricultural activities, the level of its use in South Africa is very low. Furthermore, to increase the popularity of insurance, the Act on Crop Insurance should be introduced, which will assume the use of premium subsidies for signing insurance contracts against the risk of occurrence of adverse events and their effects on agriculture. Moreover, researchers may also consider conducting their research on the willingness of other stakeholders such as financial institutions, governments and insurance companies to participate in index-based crop insurance.
